# Phylogenetic analysis of vitamin B12-related metabolism in *Mycobacterium tuberculosis*

**DOI:** 10.3389/fmolb.2015.00006

**Published:** 2015-03-04

**Authors:** Douglas B. Young, Iñaki Comas, Luiz P. S. de Carvalho

**Affiliations:** ^1^Division of Mycobacterial Research, MRC National Institute for Medical ResearchLondon, UK; ^2^FISABIO Public HealthValencia, Spain

**Keywords:** *Mycobacterium tuberculosis*, phylogenetic analysis, vitamin B12, metabolism, mycobacteria

## Abstract

Comparison of genome sequences from clinical isolates of *Mycobacterium tuberculosis* with phylogenetically-related pathogens *Mycobacterium marinum*, *Mycobacterium kansasii*, and *Mycobacterium leprae* reveals diversity amongst genes associated with vitamin B12-related metabolism. Diversity is generated by gene deletion events, differential acquisition of genes by horizontal transfer, and single nucleotide polymorphisms (SNPs) with predicted impact on protein function and transcriptional regulation. Differences in the B12 synthesis pathway, methionine biosynthesis, fatty acid catabolism, and DNA repair and replication are consistent with adaptations to different environmental niches and pathogenic lifestyles. While there is no evidence of further gene acquisition during expansion of the *M. tuberculosis* complex, the emergence of other forms of genetic diversity provides insights into continuing host-pathogen co-evolution and has the potential to identify novel targets for disease intervention.

Strains belonging to the *Mycobacterium tuberculosis* complex evolved by clonal expansion from a common ancestral population shared with *Mycobacterium canettii*, an occasional human pathogen closely resembling *M. tuberculosis* in terms of disease and evolution. *M. tuberculosis* and *M. canettii* share close phylogenetic links to other pathogens including *Mycobacterium leprae*, *Mycobacterium marinum* [and the derived *Mycobacterium ulcerans* (Doig et al., 2012)], and *Mycobacterium kansasii* (Figure 1) (Cole et al., 1998; Stinear et al., 2008; Supply et al., 2013; Blouin et al., 2014). There was an early division of the *M. tuberculosis* complex into two major branches (Brosch et al., 2002). The branch characterized by a 2 kb RD9 deletion and a single nucleotide polymorphism (SNP) that inactivates pyruvate kinase (Keating et al., 2005) gave rise to two lineages associated with human tuberculosis in West Africa (referred to as *M. africanum*) and a series of animal-adapted variants including *M. bovis*, *M. orygis*, *M. microti*, and *M. pinnipedii* (Comas et al., 2013). The branch with an intact pyruvate kinase diversified into five human lineages with patterns of geographic distribution and phylogenetic coalescence consistent with their having co-evolved with populations of modern humans migrating out of Africa around the Indian Ocean and across Eurasia (Comas et al., 2013). While the animal strains often retain the ability to cause sporadic cases of human disease (Bos et al., 2014), they do not generally establish an effective transmission cycle. Similarly, while human strains of *M. tuberculosis* can be isolated from cattle lesions in low prevalence herds (Berg et al., 2009), a high prevalence of bovine tuberculosis is always associated with cattle-adapted *M. bovis* (Firdessa et al., 2012). Epidemiological evidence suggests that individual human lineages are optimized for transmission within particular ethnic groups, providing a further indication of co-evolution between host and pathogen (Gagneux et al., 2006; Fenner et al., 2013).

**Figure 1:**
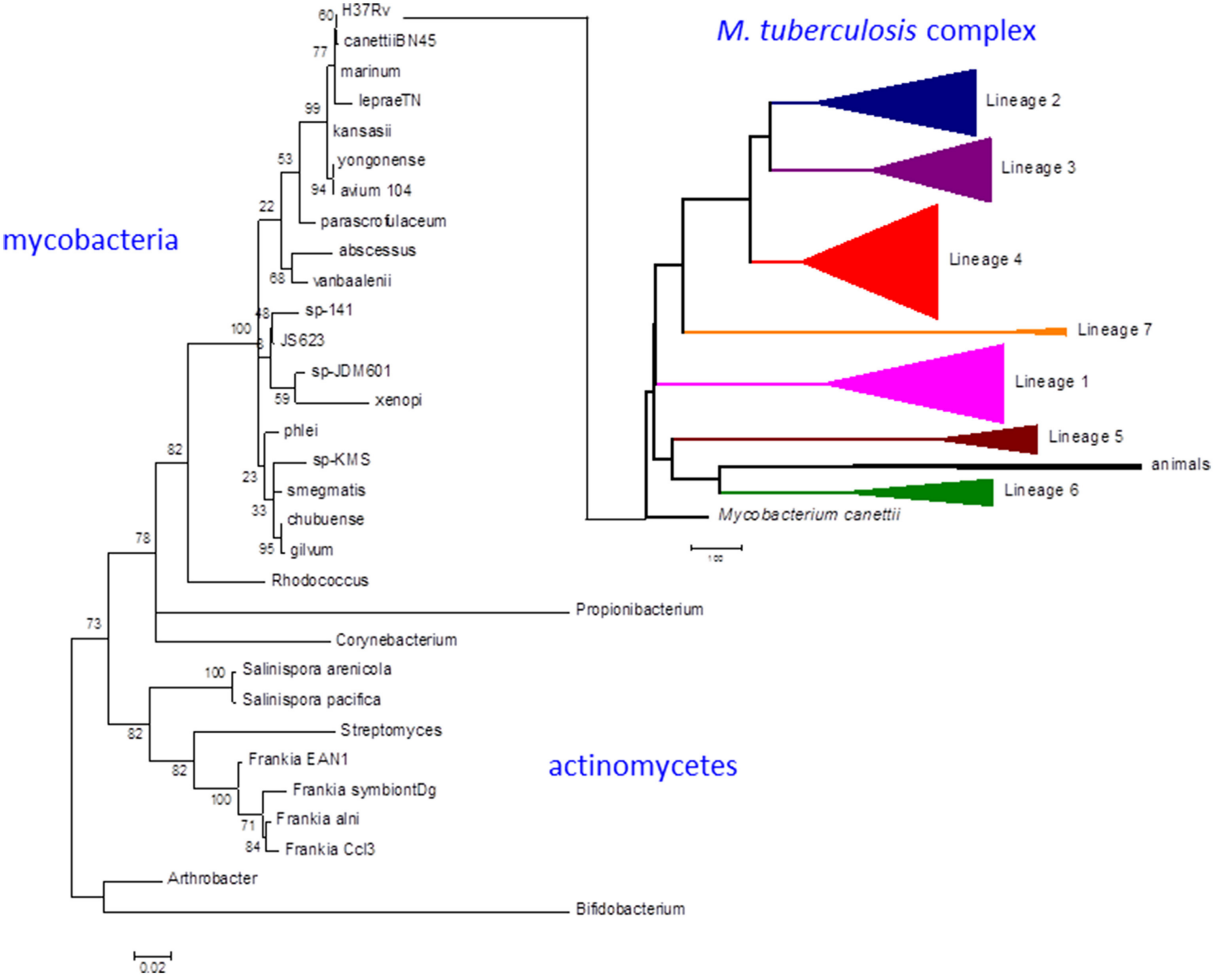
**Phylogeny of *M. tuberculosis* and related actinomycetes**. The *M. tuberculosis* complex emerged by clonal expansion of a strain closely related to the *M. canettii* cluster of slow-growing mycobacteria. The actinomycete phylogenetic tree is based on 16S rRNA sequences. Briefly, the corresponding sequences were aligned using the 16sRNA alignment tool available in the RDP database (Cole et al., 2014). The resulting alignment was analyzed with MEGA6 to infer a maximum likelihood phylogeny (Tamura et al., 2013). The best-fit model of nucleotide evolution after Akaike information criterion was Tamura-Nei with Gamma and invariants to model rate variation. Thousand bootstrap pseudo-replicates were used to give statistical support to the clades of the maximum likelihood topology. The *M. tuberculosis* complex tree is based on whole genome SNP analysis as obtained in Comas et al. (2013). Scale bar for the 16sRNA phylogeny reflects number of substitutions per site. In the case of the whole genome MTBC phylogeny reflects number of single nucleotide polymorphism. Numbers in the nodes of the 16sRNA topology reflects the percentage of bootstrap replicates supporting each node. No bootstrap support is shown for the MTBC phylogeny as all the case were higher than 95% as shown in Comas et al. (2013).

Despite sustained efforts to deliver optimal therapy over the last two decades, tuberculosis remains a major global health problem. Treatment of clinical disease undoubtedly saves lives and reduces suffering, but there are limitations to its effectiveness as a strategy to block transmission and there is a need for vaccines and preventive therapies that will arrest the disease process prior to development of an infectious state (Dye et al., 2013). Identification of molecular determinants that influence the transmission efficiency of the different host-adapted genotypes may point the way to novel interventions targeted toward disease control at a population level. It is anticipated that genetic changes that have a functional impact on pathogenesis will influence the repertoire of molecules that interact with the host immune system and the metabolic networks that support growth and survival in particular environmental niches. In this review we investigate the potential of combining phylogenetic and metabolic approaches to dissect events in the evolution of *M. tuberculosis*.

## Biosynthesis of vitamin B12

**Figure 2:**
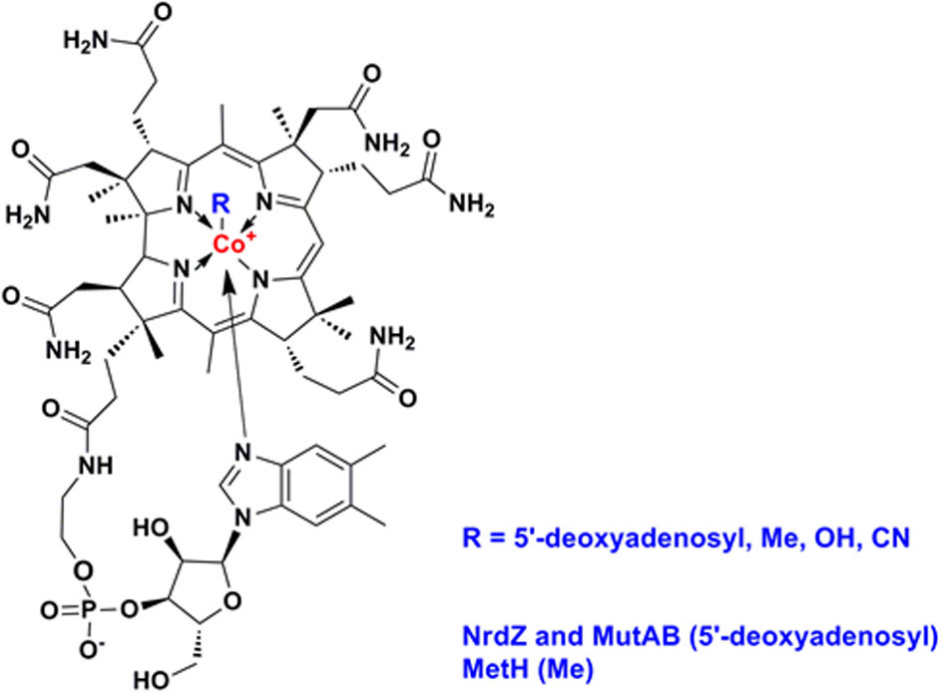
**Structure of vitamin B12 (cobalamin)**.

Vitamin B12 (also known as cobalamin, Figure 2) refers to a family of cobalt-containing, water soluble vitamins which are required in several, unrelated metabolic pathways. Due to the reactivity of its carbon-cobalt bond, B12-dependent enzymes are able to catalyze isomerizations, methyltransfers, and dehalogenations. Vitamin B12 presents an interesting focus for exploration of the metabolic phylogeny of host-pathogen interactions. It is likely that the progenitor eukaryotic cell included B12-dependent enzymes but lacked the complex set of genes required for B12 biosynthesis. Only bacteria and archaea have the machinery to synthesize vitamin B12 (Zhang et al., 2009; Doxey et al., 2015). While animals continued to use B12-dependent enzymes for methionine synthesis and methylmalonate metabolism, land plants and fungi rely on B12-independent enzymes for these pathways. Divergent evolution of algae generated both B12-dependent and B12-independent species (Helliwell et al., 2011). A fundamental feature inherent in the choice of a B12-dependent lifestyle is that it involves commitment to symbiosis with B12-producing bacteria, establishing a platform for the subsequent evolution of pathogens. Many prokaryotes have also elected to rely on community-acquired B12; bioinformatic analysis of whole genome sequences identifies an intact B12 biosynthesis pathway in only half of the bacteria that have B12-dependent enzymes (Zhang et al., 2009). Supply and utilization of vitamin B12 by bacteria can have an important impact on the metabolism of eukaryotic partners in co-culture (Wang et al., 2014; Watson et al., 2014). While it is unlikely that the relatively small numbers of tuberculosis bacteria will influence overall B12 levels in infected individuals, vitamin B12 produced or consumed by mycobacterial pathogens may have a local effect on the metabolic environment of human granulomatous lesions.

Mycobacteria and related actinomycetes generally retain the ability to synthesize B12, predominantly by the aerobic pathway, and *Propionibacterium shermanii* is used for production of the vitamin (Martens et al., 2002). More distantly related actinomycetes such as *Arthrobacter* and *Bifidobacteria* lack a B12 pathway, though some *Arthrobacter* have a B12-dependent methionine synthase. As highlighted by Boritsch et al. (2014) distinctive feature of *M. tuberculosis* is that the *cobF* gene—encoding a precorrin-6a synthase required for B12 synthesis—was deleted during evolution from the *M. canettii*-like ancestor, along with two further genes and N- and C-terminal portions of flanking genes Rv0943c and Rv0944 (Supply et al., 2013). While this may not entirely ablate B12 biosynthesis [it has been suggested that alternative methyltransferases might partially compensate for the loss of CobF (Rodionov et al., 2003; Gopinath et al., 2013a)], it suggests that *M. tuberculosis* may have come to rely on the host environment as a source of vitamin. Although classically, NrdZ and MutAB utilize adenosylcobalamin and MetH employs methylcobalamin, cofactor specificity for mycobacterial enzymes has not been investigated. This is important in all organisms that might synthesize one form of the cofactors and uptake another. Importantly, species specific variation in these cofactors, such as differences in the exact structure of the lower ligand, could indicate that mycobacterial enzymes must have greater ability to accommodate more than one form of the cofactor. Similarly, while non-typhoidal *Salmonellae* retain a B12 pathway, this is inactivated in human-adapted typhoidal serovars (Gal-Mor et al., 2014; Nuccio and Baumler, 2014), and *Yersinia pestis* and *Yersinia pseudotuberculosis* lack most of the B12 biosynthesis genes present in *Yersinia enterocolitica*. The RD9 deletion causes further attrition of the B12 pathway, removing the amino terminus of CobL, with likely polar effects on expression of CobM and CobK, and a predictive algorithm based on primary sequence conservation (SIFT; Ng and Henikoff, 2003) identifies three SNPs that are likely to impair cobalamin biosynthesis enzymes in Lineage 5 isolates (CobM D53G, CobO Q202R, CobU H50R). In the case of CobN/Rv2026c, screening a panel of more than 200 clinical isolates representative of the global diversity of *M. tuberculosis* (Comas et al., 2013) identifies 17 SNPs predicted to have an impact on protein function, two strains with >1 kb deletion, and truncated proteins generated by frameshift events. Historical experience suggests that the incidence of active tuberculosis is markedly reduced in the context of pernicious anemia (B12-deficiency) (Barron, 1933), and analysis of recent data shows a slight increase in the risk of tuberculosis during the first year after treatment for pernicious anemia (Ramagopalan et al., 2013). These clinical findings are consistent with a model in which cobalamin availability is a limiting factor for the growth of *M. tuberculosis in vivo.* Several studies have highlighted a complex effect of nutritional factors on susceptibility to tuberculosis, including a potential links to low B12 availability in vegetarian diets (Chanarin and Stephenson, 1988; Cegielski and McMurray, 2004). The obligate pathogen *M. leprae* has a severely reduced genome (Cole et al., 2001) lacking all of the B12 pathway with the exception of a set of genes required for scavenging exogenous B12 precursors (Rodionov et al., 2003; Zhang et al., 2009; Gopinath et al., 2013a): *cobT* (ML0868), ML1149/Rv1314c (ATP:cobalamin adenosyltransferase), and the BacA transporter ML2084/Rv1819c (Gopinath et al., 2013b). The B12 pathway is intact in *M. marinum* and *M. kansasii*.

These mutations suggest that *M. leprae* has fully adapted to reliance on host-derived B12, that *M. tuberculosis* is at least partially reliant on the host, and that *M. marinum* and *M. kansasii* synthesize their own B12. We next screened for diversity in the three pathways that utilize B12-dependent enzymes. In each case the bacteria have the choice of favoring the B12-dependent pathway or a parallel B12-independent mechanism.

## Biosynthesis of methionine

### B12-dependent methionine synthesis

**Figure 3:**
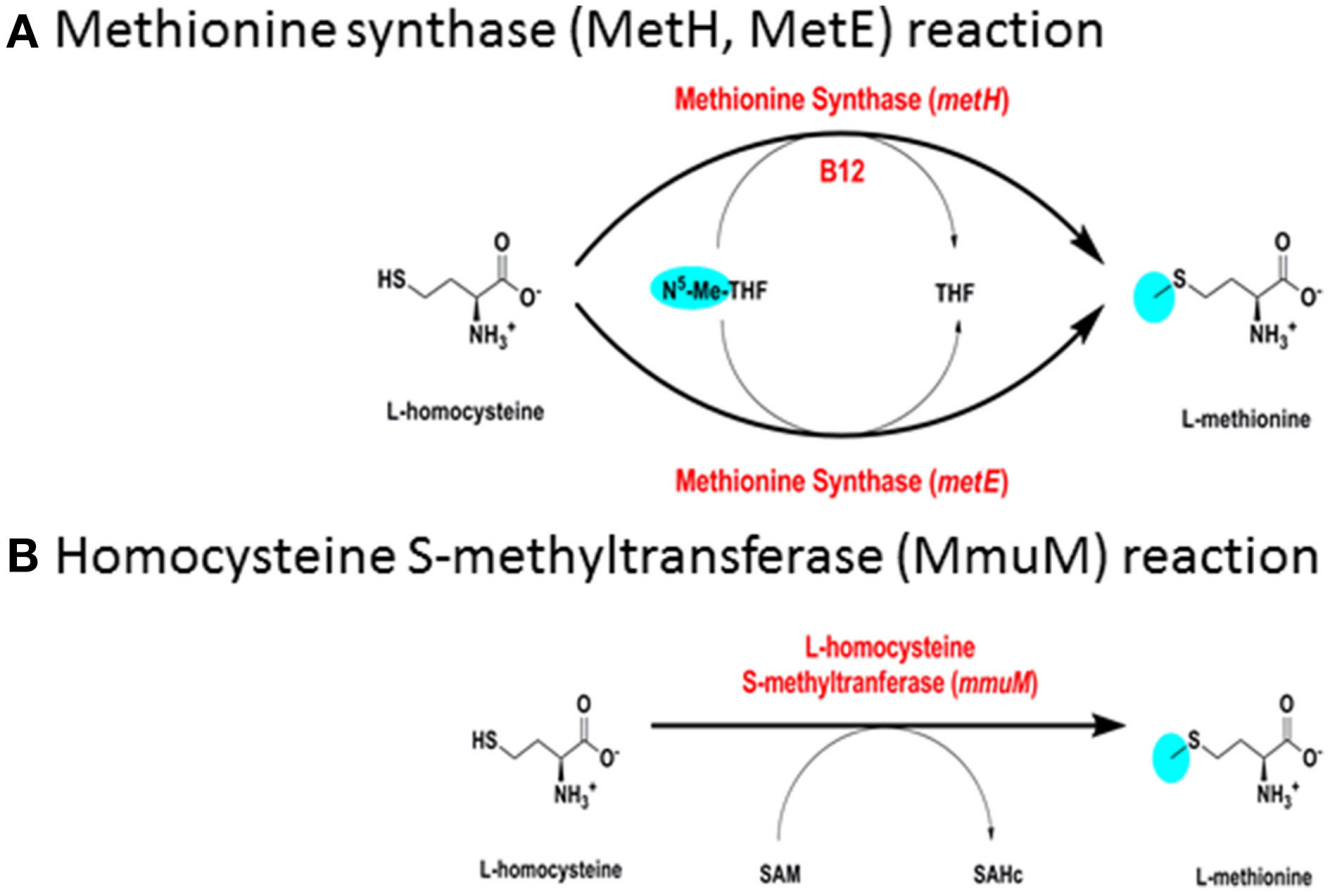
**Biosynthesis of methionine. (A)** Methionine synthase (MetH, MetE) reaction. **(B)** Homocysteine S-methyltransferase (MmuM) reaction.

**Figure 4:**
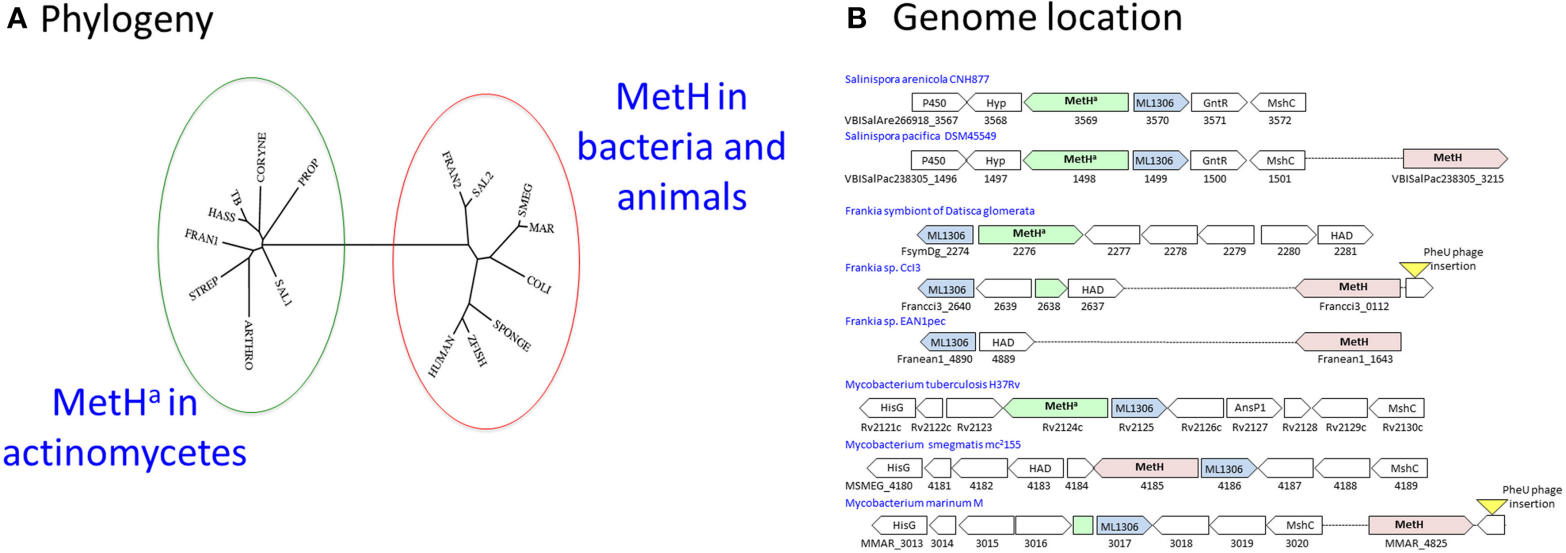
**MetH switching in actinomycetes. (A)** Phylogeny: The MetH^a^ protein of *M. tuberculosis* (TB) is similar to that commonly found in actinomycetes including *Streptomyces* (STREP), *Corynebacteria* (CORYNE), *Propionibacteria* (PROP), and *Arthrobacter* (ARTHRO). In rapid-growing mycobacteria such as *M. smegmatis* (SMEG) MetH is related to the conventional variant found in other bacteria and animals. *M. hassiacum* (HASS) is an example of a rapid-growing mycobacterium with the MetH^a^ variant, while slow-growing *M. marinum* (MAR) has MetH. One or both variants are found in *Frankia* (FRAN1/2) and *Salinispora* (SAL1/2) isolates. The phylogenetic tree was generated using the phylogeny.fr webserver (Dereeper et al., 2008). **(B)** Genome location: MetH^a^ is always located adjacent to the actinomycete signature protein ML1306. Isolates of *Salinispora pacifica* include a second MetH variant in a separate location. Acquisition of a MetH variant in *Frankia* species is associated with deletion of MetH^a^. In *M. marinum* and *Frankia sp.* CcI3, the ML1306 site contains a fragment of MetH^a^; in both species an intact conventional MetH is present adjacent to a prophage insertion into tRNA-Phe-GAA, suggesting its possible introduction by horizontal transfer. Most rapid-growing mycobacteria have a MetH variant at the ML1306 site.

*M. tuberculosis* has a B12-dependent methionine synthase (Rv2124c) with sequence characteristics matching the predominant form of MetH found in most actinomycetes (MetH^a^). MetH catalyzes the B12-dependent synthesis of L-methionine (EC 2.1.1.13), utilizing L-homocysteine and 5-methyltetrahydrofolate (N^5^-MeTHF) as substrates and generating tetrahydrofolate (THF) as a co-product (Figure 3A). While MetH^a^ shares key catalytic residues and structural features with the common form of MetH found in most bacteria and animals, sequence alignment—30% identity with *E. coli* and 29% identity with human MetH—shows it to have a distinct phylogenetic origin (Figure 4A). MetH^a^ is always located on the chromosome adjacent to a gene with homology to ML1306 (Rv2125 in *M. tuberculosis*), one of 24 conserved “signature proteins” that define the *Actinobacteria* phylum (Gao and Gupta, 2012). The function of the ML1306 proteins is unclear. Primary sequence analysis and structural modeling suggest some similarity to chaperones involved in assembly of the archaeal proteasome, but functional studies in *Streptomyces* demonstrate a role in chromosome segregation (Ditkowski et al., 2010), and structural analysis of the paralogous protein Rv2714 (another actinobacteria signature protein) revealed features reminiscent of purine nucleoside phosphorylases, carboxypeptidases, and bacterial peptidyl-tRNA hydrolases (Grana et al., 2009).

Some actinomycetes have acquired an additional *metH* gene resembling the common bacterial variant. Members of the genus *Salinispora* are obligate marine actinomycetes associated with sea sponges (Ziemert et al., 2014). While all of the sequenced *Salinispora* genomes have an ML1306-linked MetH^a^, the majority of *S. pacifica* isolates have an additional second MetH located elsewhere on the genome (Figure 4B). *Frankia* are actinomycete symbionts of plants (Tisa et al., 2013). While the obligate *Frankia* symbiont of *Datisca glomerata* has a typical actinomycete MetH^a^, other *Frankia* genomes have an alternative variant. Genome comparisons indicate that the original *metH^a^* gene was lost in most *Frankia* species as part of a 2.5 kb deletion between the ML1306 homolog and a conserved gene encoding an enzyme belonging to the haloalkanoic dehalogenase (HAD) superfamily, leaving only the acquired MetH variant (Figure 4B).

*Mycobacterium marinum* (Stinear et al., 2008) resembles *Frankia* sp. CcI3 in having an alternative MetH (MMAR_4825) with only a residual fragment of the actinomycete *metH^a^* sequence found adjacent to ML1306 homolog (MMAR_3107) (Figure 4B). In most rapid-growing mycobacteria, as well as in members of the *Mycobacterium avium-intracellulare* complex, the ML1306 site is occupied by a non-actinomycete MetH (Figure 4B). Exceptions include rapid-growing *M. hassiacum* and *M. thermoresistibile* which have a MetH^a^ similar to *M. tuberculosis*, *M. canettii*, *M. kansasii*, *M. xenopi*, and *M. leprae*.

Comparison of genome sequences from clinical isolates reveals extensive MetH^a^ polymorphism within the *M. tuberculosis* complex. Isolates from the sub-branch of EuroAmerican Lineage 4 that includes the well-characterized strain CDC1551 have a deletion that generates a non-functional MetH^a^ (Warner et al., 2007). A further 20 non-synonymous SNPs spread throughout the protein are predicted to have an effect on enzyme function (Table 1). With the exception of E220D in Lineage 5, these SNPs occur close to the tips of the phylogenetic tree affecting only one or a few isolates and suggesting that they are of relatively recent origin. The frequent occurrence of functional SNPs—in comparison to MetE, for example—is consistent with the proposal of Warner et al. (2007) that loss of MetH^a^ might have some selective benefit.

**Table 1:** ***M. tuberculosis* MetH^a^ SNPs with predicted functional impact**.

**Position**	**Ancestral**	**Derived**	**Mutation**	**SIFT**	**Isolates**
2386028	G	A	L14F	0.03	L6_N0090, L6_414104
2385793	A	C	I92S	0.00	L2_M4100A
2385695	C	T	G125R	0.00	L4_155008, L4_GM1503, L4_N0146, L4_N0137, L4_X632
2385408	C	A	E220D	0.00	Lineage 5
2385338	G	C	H244D	0.00	L3_N0054
2385283	G	A	P262L	0.00	L4_V639EA
2385181	C	T	G296D	0.00	L1_T17
2385146	C	T	V308M	0.01	L6_N0060
2384545	A	G	I508T	0.03	L3_751B
2384452	C	T	R539H	0.02	L2_N0017, L2_981833
2384402	G	T	L556I	0.03	L6_538302
2384359	T	C	H570R	0.01	L7_BTBH935
2384009	T	C	T687A	0.04	L4_N0163
2383951	G	T	A706E	0.00	L1_N1004
2383903	C	T	R722H	0.00	L3_N1058
2383616	C	T	G818S	0.00	L3_162908, L3_155808, L3_159008
2383051	A	C	I1006S	0.00	L4_478304
2382954	G	T	F1038L	0.00	L1_N72, L1_N70, L1_N0182, L1_N0203
2382776	A	C	Y1098D	0.00	L2_N0010, L2_N0130, L2_N1037, L2_N0020, L2_N005, L2_N0150
2382623	G	C	R1149G	0.02	L6_N0090, L6_414104
2382501	G	C	Y1189X		L4_N0148, L4_N1057
2383616	2382501		Deletion		L4_CDC1551, L4_DY22, L4_N0142, L4_X581

Screening of a global diversity panel of M. tuberculosis isolates identified 25 non-synonymous SNPs in MetH^a^/Rv2124c; 21 of these are predicted by the SIFT algorithm (Ng and Henikoff, 2003) to have an impact on enzyme function. A similar screen for MetE/Rv1133c identified 3 SNPs, only one of which is predicted to affect function (L6_N0092). Strain numbers refer to Comas et al. (2013).

### B12-independent methionine synthesis

*M. tuberculosis* has a putative B12-independent methionine synthase (EC 2.1.1.14), Rv1133/MetE, similar to that found in a wide range of bacteria. MetE catalyzes the B12-independent synthesis of L-methionine from L-homocysteine (Figure 3A), with N^5^-MeTHF as the methyl donor, similarly to MetH. A B12-independent reaction (EC 2.1.1.10) can also be achieved by transfer of a methyl group from S-adenosylmethionine by homocysteine S-methyltransferase (Rv2458/MmuM), as shown in Figure 3B. Rv2458 encodes an MmuM homolog in *M. tuberculosis* but does not substitute for MetE activity in a MetH knockout background (Warner et al., 2007). This is likely a reflection of the co-substrate needed in the reaction catalyzed by MmuM; S-adenosylmethionine versus N^5^-MeTHF utilized by MetH and MetE. As S-adenosylmethionine is synthesized by condensation of methionine and ATP it is impossible for this pathway to supply enough methionine for physiologic demands, as it will always consume one equivalent of S-adenosylmethione per methionine made, which in turn needs methionine to be regenerated. MmuM is conserved in the reduced genome of *M. leprae* but is absent in rapid-growing mycobacteria and has been deleted from *M. marinum*. In common with other actinomycetes, *M. tuberculosis* has a predicted protein with sequence match to the C-terminal catalytic domain of MetE (Rv3015c) that is often annotated as a MetE homolog. However, the canonical MetE enzyme has a duplicated domain structure (Pejchal and Ludwig, 2005) and the functional activity of the single domain proteins is unclear.

**Figure 5:**
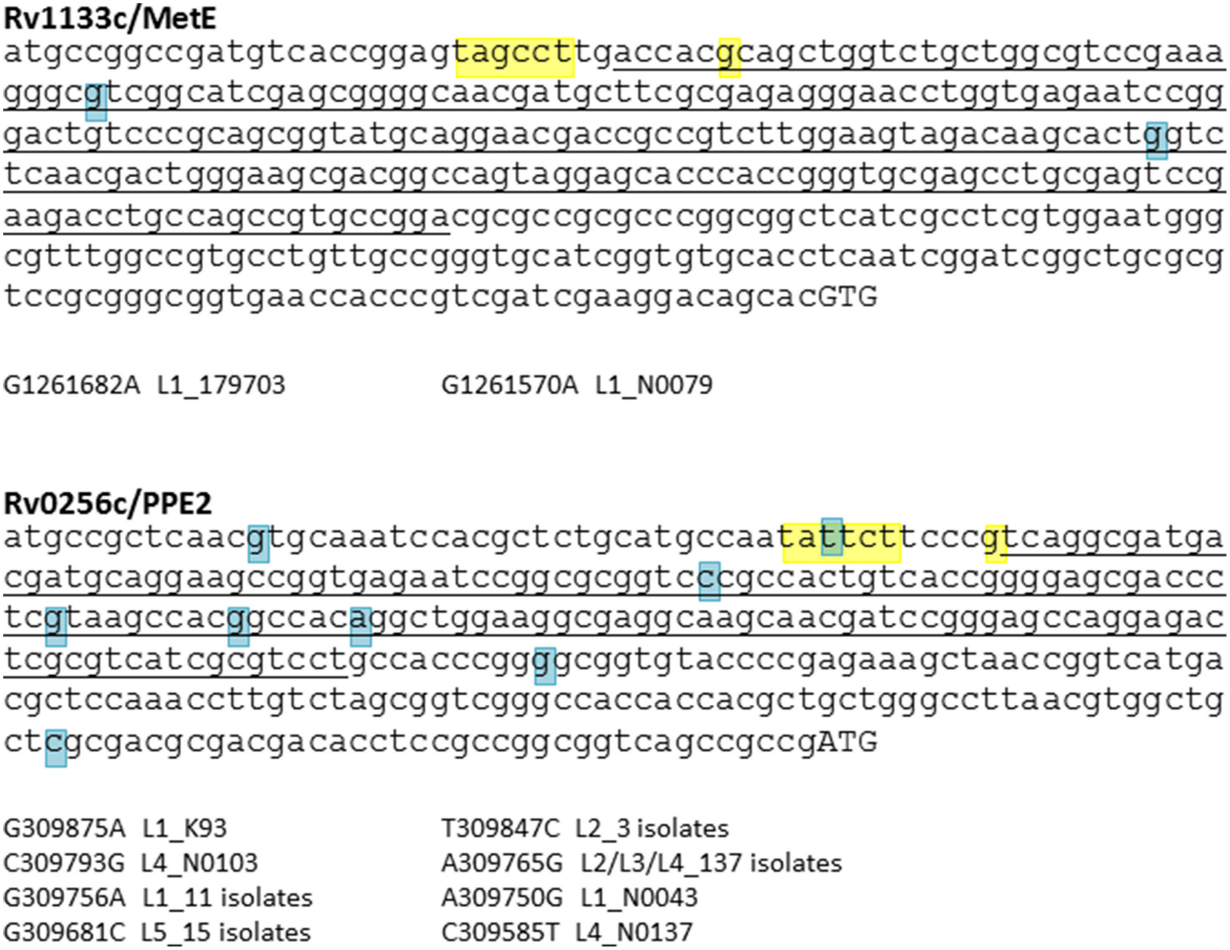
**SNP analysis of B12 riboswitches**. *M. tuberculosis* has two B12-dependent riboswitches, located upstream of Rv1133c (MetE) and Rv0256c (PPE2). Riboswitch sequences identified by Vitreschak et al. (2003) are underlined, −10 motifs and transcription start sites (Cortes et al., 2013) are highlighted in yellow, and start codons in uppercase. Blue highlights show SNPs identified in a panel of clinical isolates representative of the global diversity of *M. tuberculosis* (see Comas et al., 2013, for details of isolates). While there is evidence of diversification of the Rv0256c riboswitch, the MetE riboswitch has been conserved during expansion of the *M. tuberculosis* complex.

The catalytic turnover rate of *E. coli* MetH is 100-fold higher than MetE (Gonzalez et al., 1992), conferring a clear metabolic and ecological advantage in use of the B12-dependent pathway when B12 is available. Equivalent kinetic and thermodynamic constants for mycobacterial MetH, MetH^a^, and MetE are unknown; their determination might provide a functional rationale for MetH switching events. Expression of MetE in both *E. coli* and *M. tuberculosis* is repressed in the presence of B12 by binding of the cofactor to a regulatory riboswitch upstream of the *metE* coding sequence (Vitreschak et al., 2003; Warner et al., 2007). Warner et al. showed that *in vitro* culture of MetH^a^-negative *M. tuberculosis* in the presence of exogenous B12 resulted in selection of mutations in the MetE riboswitch (Warner et al., 2007), but there is no evidence of equivalent SNPs in clinical isolates with MetH^a^ deletions or mutations (Figure 5).

In contrast to MetE, accumulation of SNPs is observed in the case of a second *M. tuberculosis* B12 riboswitch located upstream of PPE2/Rv0256c (Vitreschak et al., 2003; Warner et al., 2007) (Figure 5). The function of Rv0256c is unclear. The presence of the riboswitch, together with a predicted membrane-spanning structure and potential operon link to B12 biosynthesis genes, led to a suggested role in cobalt transport (“CbtG”) (Rodionov et al., 2003). A recent elegant genetic study identified the BacA transporter as the sole determinant of cobalamin uptake by *M. tuberculosis* (Gopinath et al., 2013b). Riboswitch-regulated Rv0256c is retained in other mycobacteria including *M. leprae*, though the operon link to *cob* genes is variable.

During exponential growth of *M. tuberculosis* H37Rv, *metE* and *metH^a^* transcripts are present at a similar abundance around 5-fold higher than the median for all genes (Cortes et al., 2013). In terms of protein abundance, however, MetE is in 100-fold excess compared to MetH^a^ (Cortes et al., 2013), suggesting that the MetH^a^ protein is either translated less efficiently, or is more readily degraded. Proclivity to degradation might be a consequence of poor stability of MetH in the absence of B12, and it can be anticipated that addition of vitamin B12 to the growth medium might alter the protein ratio. In *M. marinum*, the *metH* transcript has an abundance matching the median level for all genes, with *metE* transcript 10-fold lower (Wang et al., 2013). This is consistent with the notion that contrary to *M. tuberculosis*, *M. marinum* constitutively produces B12.

### Methionine and folate metabolism

The methyl group for the MetH/MetE reaction is provided by N^5-MeTHF^, and imbalance of vitamin B12 metabolism in humans is often associated with imbalance in folate metabolism. Similarly, disruption of methionine levels is observed in para-aminosalicylic acid treated *M. tuberculosis* (Chakraborty et al., 2013), indicating a tight coupling between folate biosynthesis and methionine levels. N^5^-MeTHF is generated from 5,10-methylenetetrahydrofolate by the MetF reductase, and mycobacteria are unusual amongst the actinomycetes in lacking an annotated copy of MetF. An exception is *Mycobacterium abscessus*, in which a MetF homolog is present between genes encoding lipoprotein LppM and geranylgeranyl pyrophosphate synthase IdsA. Other mycobacteria have a conserved hypothetical in this location; Rv2172c in *M. tuberculosis*. While Rv2172c shows no significant primary sequence homology with MetF, fold-recognition and structural modeling (Kelley and Sternberg, 2009) reveals a significant match with the crystal structure of MetF from *Thermus thermophilus*, identifying Rv2172c as an atypical MetF homolog.

## Propionyl-CoA catabolism

### Methylmalonate pathway (B12-dependent)

Toxicity linked to the accumulation of propionyl-CoA derivatives during catabolism of odd-chain fatty acids is countered by their metabolism through either the B12-dependent methylmalonate pathway or the B12-independent methylcitrate cycle. One or other of the two pathways is essential for growth of *M. tuberculosis* on propionate (Munoz-Elias et al., 2006; Savvi et al., 2008; Eoh and Rhee, 2014). Methylmalonyl-CoA is also an important substrate for biosynthesis of cell wall lipids (Rainwater and Kolattukudy, 1983, 1985; Azad et al., 1997; Fernandes and Kolattukudy, 1998).

**Figure 6:**
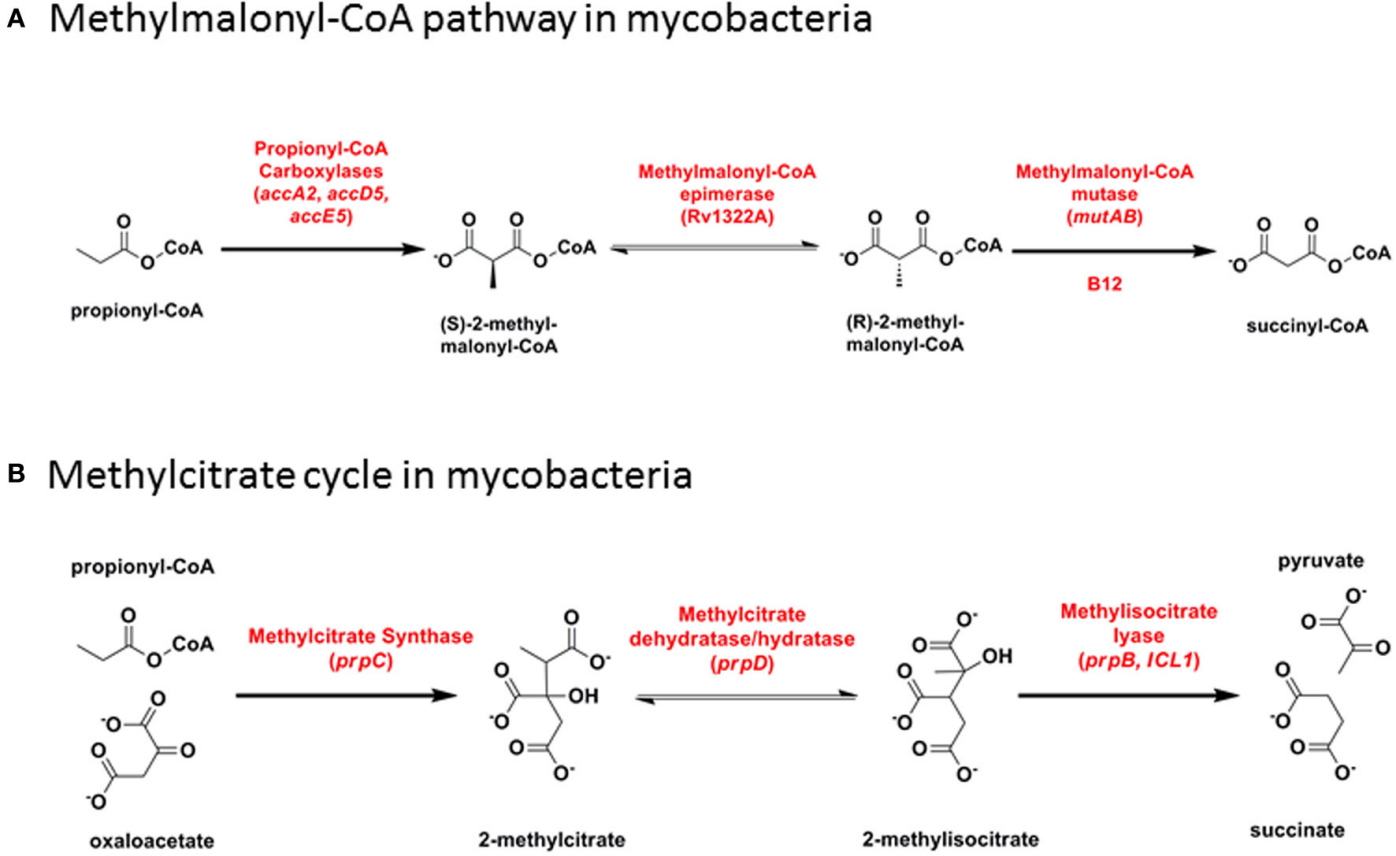
**Propionyl-CoA catabolism. (A)** Methylmalonyl-CoA pathway in mycobacteria. **(B)** Methylcitrate cycle in mycobacteria.

The methylmalonate pathway (Figure 6A) including B12-dependent MutAB methylmalonyl-CoA mutase (EC 5.4.99.2) is conserved across all mycobacteria including *M. leprae*. Propionyl-CoA enters the methylmalonate pathway through an essential acyl-CoA carboxylase comprising alpha (AccA3), beta (AccD5), and epsilon (AccE5) subunits (Gago et al., 2006; Lyonnet et al., 2014). Rv3281, encoding the epsilon subunit, is highly polymorphic between mycobacterial species and across multiple *M. tuberculosis* isolates, with a predicted N-terminal domain consisting of a variable number of repeat elements generating protein products that range from 85 to 203 amino acids. Functional activity of the epsilon subunit is retained in a 76 amino acid fragment from the conserved C-terminus [using valine-122 as start codon (Gago et al., 2006)] and the role—if any—of the variable N-terminus is unclear. Shotgun proteome analysis of *M. tuberculosis* H37Rv detected five peptides spanning the sequence from valine-63 to the C-terminus (Cortes et al., 2013) showing that at least part of the variable N-terminus is translated into protein.

Mycobacterial Rv1492/MutA and Rv1493/MutB are presumed to form an alpha-beta heterodimer structure analogous to the well-characterized B12-dependent methylmalonyl-CoA mutase from *Propionibacterium shermanii* (Cracan and Banerjee, 2012); there is no evidence of diversification during the evolution of *M. tuberculosis*. *M. canettii*, and hence *M. tuberculosis*, lacks a locus containing a second mycobacterial methylmalonyl-CoA mutase (McmA2, MMAR_4797/4798 in *M. marinum*). This is a split-domain enzyme with substrate and B12 cofactor binding sites located on separate polypeptides. McmA2 is located in a ~150-gene region between Rv0838/LpqR and Rv0852/FadD16 that includes six conserved cytochrome P450 genes and an MCE locus. *M. tuberculosis* has only 15 genes in this region, *M. leprae* has eight genes and seven pseudogenes, and a 50-gene deletion removes the MCE locus and three of the P450s in *M. ulcerans*. The presence of a series of lipid-related enzymes in the MmcA2 region suggests that the role of the mutase in this case may be to generate methylmalonyl-CoA units for biosynthesis of a secondary metabolite or complex lipid that is missing from *M. tuberculosis.*

### Methylcitrate cycle (B12-independent)

In the methylcitrate cycle (Figure 6B), 2-methylcitrate synthase (PrpC) combines propionyl-CoA with oxaloacetate to form 2-methylcitrate (EC 2.3.3.5), which is converted to methylisocitrate by PrpD dehydratase/hydratase (EC 4.2.1.79 and 4.2.1.99). These two steps resemble the first two reactions in the Krebs cycle, catalyzed by citrate synthase and aconitase, respectively. Methylisocitrate lyase (MCL, PrpB) converts methylisocitrate to pyruvate and succinate (EC 4.1.3.30) in a reaction analogous to that catalyzed by isocitrate lyase (ICL) in the glyoxylate shunt. In *M. canettii*/*M. tuberculosis* and in *M. kansasii*, a deletion between PrpD/Rv1130 and PrpC/Rv1131 results in loss of MCL. Differences in the length of residual intergenic sequence in the two species—54 bp in *M. tuberculosis* and 130 bp in *M. kansasii*—suggests that this may have occurred through two independent events. The methylcitrate cycle remains fully functional in *M. tuberculosis*, however, with ICL catalyzing the final step (Munoz-Elias et al., 2006; Eoh and Rhee, 2014). MCL is also disrupted by a frameshift mutation in *M. ulcerans* (Doig et al., 2012).

**Figure 7:**
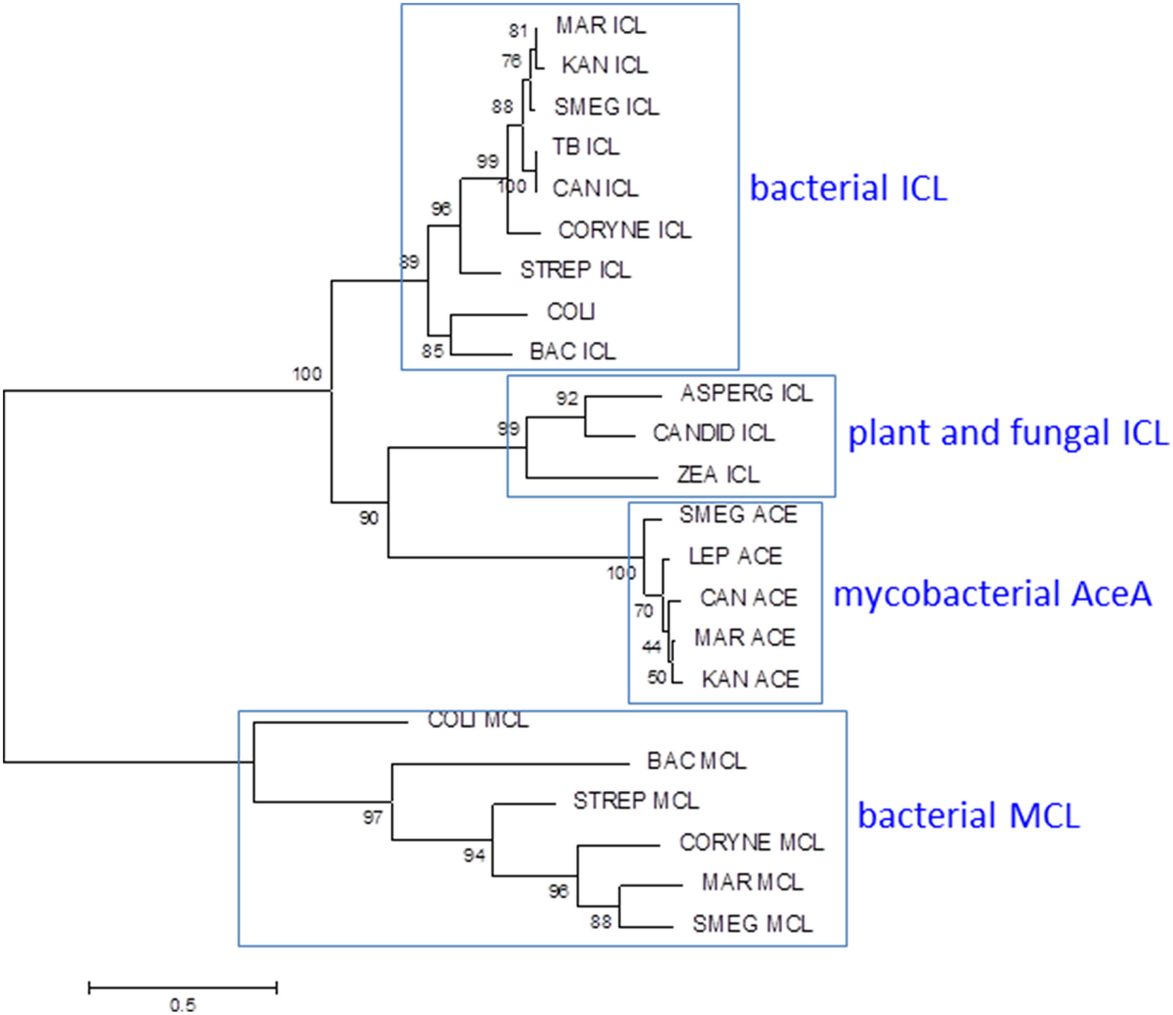
**Phylogenetic analysis of mycobacterial isocitrate lyase (ICL) and 2-methylisocitrate lyase (MCL)**. Most mycobacteria have ICL and MCL enzymes similar to those found in other bacteria. MCL has been deleted from *M. tuberculosis* and *M. kansasii* and *M. leprae* lacks both MCL and ICL1. Mycobacteria also have a second isocitrate lyase (AceA) with a distinct phylogenetic origin. The ICL/MCL phylogeny was obtained after homolog searching of the ICL and MCL proteins present in *M. tuberculosis* and *M. marinum*. Muscle was used for amino acid alignment and a maximum likelihood phylogeny inferred as implemented in MEGA6 (Tamura et al., 2013). The JTT model of amino acid substitution incorporating gamma and invariant categories for rate variation was used. Thousand bootstrap pseudoreplicates were analyzed to obtain statistical support for the clades observed in the maximum likelihood topology.

Mycobacteria have two ICLs. Icl1 (Rv0467 in *M. tuberculosis* H37Rv) is a typical bacterial ICL, clustering with related enzymes from other actinomycetes, Gram-positive and Gram-negative bacteria (Figure 7). The second ICL—generally annotated as AceA (Rv1915/Rv1916 in H37Rv)—is phylogenetically distinct from other bacterial and fungal ICLs. A single nucleotide deletion causes a frameshift in the *aceA* gene of H37Rv, resulting in its expression as two separate polypeptides which are unable to complete the methylcitrate cycle in an Icl1 mutant (Munoz-Elias et al., 2006).

Evolution of MCL activity from an ICL template has been studied in fungi and relevant amino acid changes have been mapped (Muller et al., 2011). Comparison of the ICL sequence from MCL-positive *M. marinum* with those from MCL-negative *M. tuberculosis* and *M. kansasii* reveals no obvious adaptive mutations, however, suggesting that the mycobacterial ICL has an intrinsic ability to utilize either substrate (Gould et al., 2006). The primary role of ICL is to catalyze conversion of isocitrate to succinate and glyoxylate to maintain energy generation and gluconeogenesis during growth on fatty acids. It can be anticipated that assigning a dual role to ICL in the methylcitrate cycle in addition to the glyoxylate shunt may require alterations in regulatory networks. Activation of the glyoxylate shunt in response to low glucose is controlled by the transcriptional regulator RamB (Rv0465c) (Micklinghoff et al., 2009), while expression of genes involved in the methylcitrate cycle is regulated by PrpR (Rv1129c) (Masiewicz et al., 2012). Both regulators bind to overlapping sites 80 bp upstream of the *icl1* transcription start site. The binding site is conserved between MCL-positive and MCL-negative mycobacteria, however, suggesting that while there is an interplay between the two regulators, this predates the loss of MCL. Sequence comparisons within the *M. tuberculosis* complex highlight an intriguing diversity in the amino acid sequence of the RamB regulator. Four lineage-associated SNPs are predicted to have an impact on RamB function: N36D in Lineage 6, R106C in Lineage 4, Q121R in Lineage 1, and K229T in Lineage 5. Impaired function of RamB would reduce repression of ICL in the presence of glucose, potentially enhancing availability of ICL for the methylcitrate cycle. Therefore, species-specific alterations in regulatory networks further diversified by strain-specific SNPs in transcriptional regulators allow expression of an originally fatty acid catabolic pathway during growth on carbohydrates. Changes such as these are believed to confer on *M. tuberculosis* the unique ability to grow optimally with multiple types of carbon sources (de Carvalho et al., 2010; Rhee et al., 2011), which is believed to be the case during infection. Further rewiring of metabolic circuits linking glucose and fatty acid metabolism is likely to accompany the inactivation of pyruvate kinase in Lineage 5, Lineage 6, and the associated animal-adapted strains (Keating et al., 2005).

The origin and role of the second ICL in mycobacteria is unclear. While the methylcitrate cycle genes have been deleted and *icl1* and *ramB* are reduced to pseudogenes in *M. leprae*, *aceA* and malate synthase remain intact, providing the potential for a functional glyoxylate shunt. Pyruvate kinase is intact in *M. leprae*, but pyruvate carboxylase is a pseudogene.

## Biosynthesis of deoxyribonucleotides

**Figure 8:**
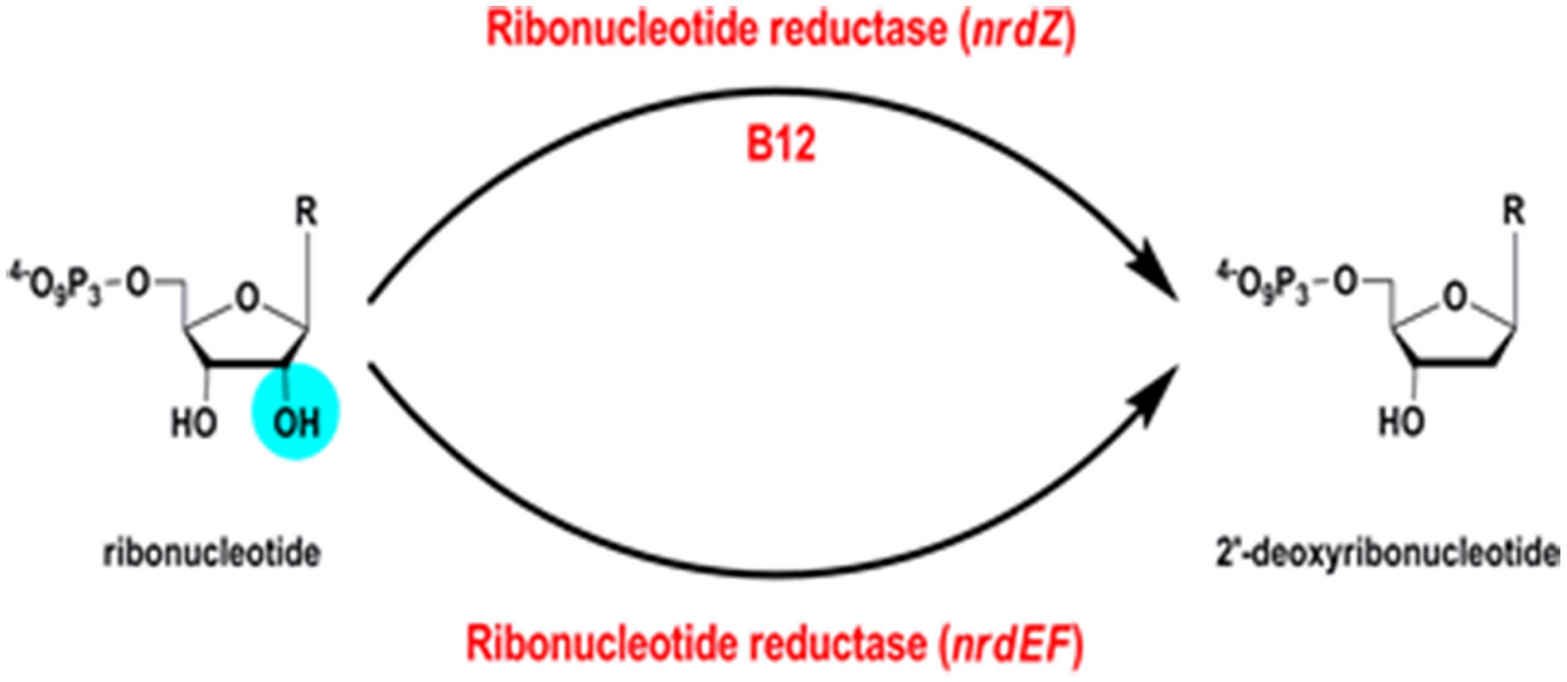
**Ribonucleotide reductase reaction**.

Ribonucleotide reductases (RNRs) are essential enzymes in all living cells, responsible for reduction of ribonucleotides to deoxyribonucleotides (EC 1.17.4.1) (Figure 8); the building blocks required for the synthesis of DNA.

### B12-dependent ribonucleotide reductase

A B12-independent class Ib ribonucleotide reductase encoded by Rv3051c/*nrdE* and Rv3048c/*nrdF2* is both necessary and sufficient to support growth of *M. tuberculosis* in a mouse model of infection (Dawes et al., 2003; Mowa et al., 2009). In addition, *M. tuberculosis* has a B12-dependent class II RNR, Rv0570 annotated as NrdZ (generally referred to as NrdJ in other bacteria). While the class I RNR requires oxygen, the class II enzyme has the potential to contribute an additional function under anaerobic conditions. In *Pseudomonas aeruginosa*, for example, a class II RNR is required to support biofilm growth under anaerobic conditions (Lee et al., 2012). It is important to note that B12 biosynthesis in mycobacteria requires oxygen, indicating that an anaerobic B12-dependent RNR would have to obtain B12 from exogenous sources under hypoxia.

Class II RNRs resemble class I and class III structurally (Sintchak et al., 2002) and the enzyme from *Lactobacillus leichmannii* is a monomer, contrasting with the oligomeric class I RNRs. In class II RNRs, the B12 cofactor is in its adenosylcobalamin form and is responsible for generation of the radical needed for the deoxygenation of the ribonucleotide. Reducing equivalents for the reaction are provided by oxidation of a pair of cysteine residues in class II RNRs (Booker et al., 1994), similarly to the mechanism described in class I RNRs.

**Figure 9:**
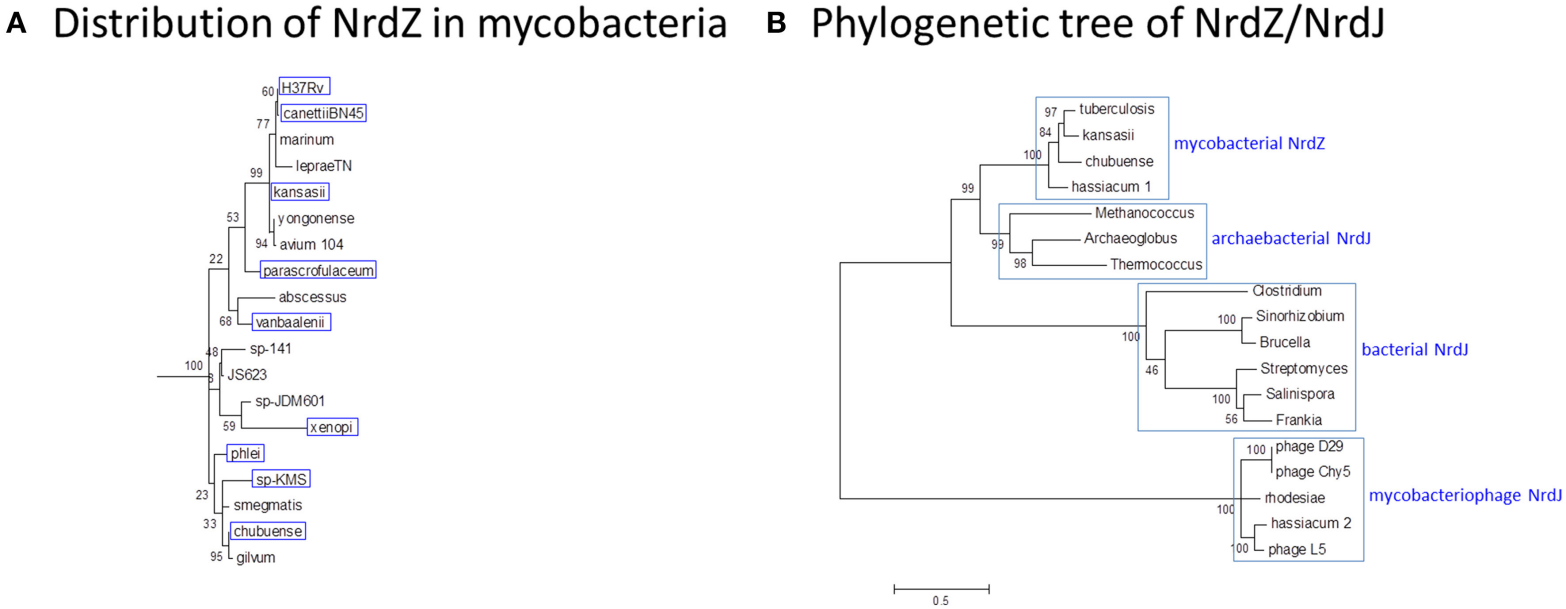
**Phylogeny of B12-dependent ribonucleotide reductase (NrdZ). (A)** Distribution of NrdZ in mycobacteria. NrdZ-positive mycobacteria are boxed in blue. The distribution of NrdZ in mycobacteria does not align with a conventional phylogeny. The mycobacteria phylogenetic tree is based on 16S rRNA sequences as described for Figure 1. **(B)** Phylogenetic tree of NrdZ/NrdJ. Mycobacterial NrdZ has a phylogenetic origin distinct from that of class II RNRs in other eubacteria, clustering more closely with archaebacterial NrdJ. Copies of NrdJ carried by mycobacteriophages and occasionally inserted into mycobacterial genomes also have a separate phylogenetic origin. The NrdZ phylogeny was obtained after homolog searching of the NrdZ protein present in *M. tuberculosis*. Muscle was used for amino acid alignment and a maximum likelihood phylogeny inferred as implemented in MEGA6 (Tamura et al., 2013). The JTT model of amino acid substitution incorporating gamma and invariant categories for rate variation was used. Thousand bootstrap pseudoreplicates were analyzed to obtain statistical support for the clades observed in the maximum likelihood topology.

NrdZ is differentially distributed amongst the mycobacteria in a pattern that is inconsistent with conventional phylogeny (Figure 9A); while it is present in slow-growing *M. tuberculosis* and *M. kansasii* and rapid-growing *M. phlei*, for example, it is absent from *M. marinum* and *M. smegmatis*. The mycobacterial class II RNR clusters separately from analogous enzymes in other actinomycetes, with a stronger resemblance to archaeal NrdJ (Figure 9B), suggesting its possible acquisition by horizontal transfer. Consistent with this, NrdJ homologs are found in the genomes of multiple mycobacteriophages (Dwivedi et al., 2013). In a phylogenetic analysis mycobacteriophage NrdZs cluster separately from chromosomal NrdZs; copies of the phage variant are integrated in the genomes of *M. hassiacum* (which also has an NdrZ) and *M. rhodesiae* JS60.

Phylogenetic analysis reveals an extensive network of horizontal exchange amongst the mycobacteria (Becq et al., 2007; Stinear et al., 2008; Veyrier et al., 2009; Supply et al., 2013; Boritsch et al., 2014; Wang and Behr, 2014). Features associated with virulence attributes in *M. tuberculosis*—including Type VII secretion and MCE loci—are commonly found in the sequence of plasmids isolated from rapid-growing mycobacteria, and genes present in multiple copies on the *M. tuberculosis* genome—cytochrome P450s, adenylate cyclases, toxin-antitoxin modules, and WhiB transcription factors—are present on plasmids, mycobacteriophages, and integrated conjugative elements (Bordeleau et al., 2012). These may provide a pool of initially unassigned functional units that can be customized for specific purpose in a particular niche, or else discarded. While horizontal acquisition made an important contribution in evolution of the common ancestor with *M. canettii*, there is little or no evidence of horizontal transfer during diversification of the *M. tuberculosis* complex. Most strikingly, in spite of strong selective pressure there is no evidence of exchange of drug-resistance determinants. Presumably the pathogenic lifestyle of *M. tuberculosis* has resulted in its isolation from opportunities for horizontal acquisition.

NrdZ is part of the DosR regulon in *M. tuberculosis*, which comprises a set of genes that are induced in response to stress stimuli sensed by a two-component regulator (Park et al., 2003). Characterization of the DosR regulon in mycobacteria and related actinomycetes has identified a set of common “core” genes encoding universal stress proteins, α-crystallin chaperones, nitroreductase-domain proteins, triacylglyceride synthases, and YfiA-like hibernation factors that are presumed to facilitate transition to a survival phenotype, together with a set of “peripheral” genes with restricted distribution (Gerasimova et al., 2011). The DosR regulon in *M. tuberculosis* is triggered by exposure to nitric oxide, carbon monoxide, or hypoxia. NrdZ is a peripheral component of the DosR regulon and may have specific relevance for hypoxic survival of *M. tuberculosis*. In *M. tuberculosis*, NrdZ is located in a 63-gene region between Rv0568/Cyp136B and Rv0629/RecD, that includes seven toxin-antitoxin modules and an *mce* locus. This region has only 17 genes in *M. marinum*, while the corresponding 61-gene region in *M. kansasii* includes NrdZ (MKAN_19005) and other genes linked to anaerobic metabolism. These anaerobic functions are missing from *M. marinum* and *M. leprae* and may have been acquired by horizontal transfer.

The NrdZ locus in *M. tuberculosis* and *M. kansasii* also includes the peripheral DosR regulon gene *pncB2* (Rv0573c, MKAN_19060) encoding a phosphoribosyltransferase involved in nicotinamide salvage. Again, PncB2 is absent from *M. marinum*. Nicotinamide provides another example of a vitamin that can be shared by host and pathogen. While *M. tuberculosis* has the potential to synthesize nicotinamide adenine dinucleotide (NAD^+^) *de novo*, growth of an *nadABC* deletion mutant in a mouse model (Vilcheze et al., 2010) shows that—in common with other human pathogens including *Shigella flexneri*, Streptococci and *Staphylococcus aureus* (Prunier et al., 2007; Sorci et al., 2013)—*M. tuberculosis* is able to scavenge nicotinamide from the host during infection. However, while *M. leprae* has preserved the capacity for *de novo* synthesis, it has lost the *pncA* and *pncB* salvage pathway genes. PncA is inactivated by an H57D SNP in cattle-adapted *M. bovis*, an L177R SNP is predicted to impact PncB2 function in Lineage 5, and PncA mutations are selected in pyrazinamide-resistant strains of *M. tuberculosis* without incurring any loss in pathogenesis, suggesting that retention of *de novo* synthesis is preferred over host scavenging as a source of the vitamin. Retention of different pathways by different pathogens may reflect selective pressures linked to nicotinamide availability in particular colonization niches.

### B12-independent ribonucleotide reductase

Genes encoding components of the major class Ib RNR—NrdE (Rv3051c) and NrdF2 (Rv3048c), with electrons supplied by NrdH (Rv3053c) and NrdI (Rv3052c)—are conserved in all mycobacteria, and additional genes with sequence homology to the thioredoxin/glutaredoxin-like NrdH are found on plasmids and mycobacteriophages (Dwivedi et al., 2013). In addition, in common with some other mycobacteria, *M. tuberculosis* has a second beta subunit gene (NrdF1/Rv1981c) and a gene encoding a third beta subunit that resembles a class Ic RNR (NrdB/Rv0233). While the alternative beta subunits are not essential for normal growth (Mowa et al., 2009), NrdF1 is active in a biochemical assay (Hammerstad et al., 2014) and it is possible that they make some contribution to survival in particular environments. The differential distribution of NrdB amongst mycobacterial species and the presence of a homolog on a plasmid from *M. yongonense* (pMyong1, OEM_p100240) suggest that it is also subject to exchange by horizontal transfer.

Expression of the class Ib RNR is regulated by transcription factor NrdR binding to characteristic tandem sites upstream of *nrdF2* and *nrdH* (Rodionov and Gelfand, 2005). Consistent with the demonstration of repressor function for NrdR (Mowa et al., 2009), the binding sites are located at positions +15.5/+47.5 (*nrdF2*) and +15.5/+60.5 (*nrdH*) with respect to transcription start sites at 3409418 and 3415168, respectively (Cortes et al., 2013). The 32 bp spacing between tandem NrdR sites upstream of *nrdH* (Rodionov and Gelfand, 2005) is extended in *M. tuberculosis* H37Rv by duplication of a 13 bp sequence. In isolates belonging to “modern” Eurasian Lineages 2, 3, and 4 and Ethiopian Lineage 7, a C/T SNP at position 3415332 generates a new TANNNT −10 consensus motif (Rose et al., 2013) and an additional transcription start site upstream of NrdH providing the potential for NrdR-independent expression.

## Utilization of ethanolamine

The ability to utilize ethanolamine is an important virulence determinant for enteric pathogens (Garsin, 2010) and *M. marinum* has *eutB* and *eutC* genes encoding a predicted B12-dependent ethanolamine ammonia-lyase. These genes are inserted along with an ethanolamine transporter and an adenylate cyclase between the end of a carbon monoxide dehydrogenase locus (MMAR_0662/Rv0368c) (Cook et al., 2014) and a conserved glycosyl hydrolase (MMAR_0667/Rv0365c). In *M. tuberculosis* this location is occupied by genes encoding a zeta-epsilon toxin-antitoxin pair (Rv0367c-Rv0366c). Amongst the slow-growing mycobacteria, the three ethanolamine utilization genes are also present in *M. gastri*, and *M. parascrofulaceum* has copies of *eutB* and *eutC*. In host-adapted *M. ulcerans*, *eutB*, the ethanolamine transporter and the adenylate cyclase are present in the form of pseudogenes. A subset of rapid-growing mycobacteria, including *M. smegmatis* mc^2^155, have the potential for ethanolamine utilization, with the relevant enzymes likely sequestered within specialized microcompartments (Axen et al., 2014).

## Concluding comments

**Figure 10:**
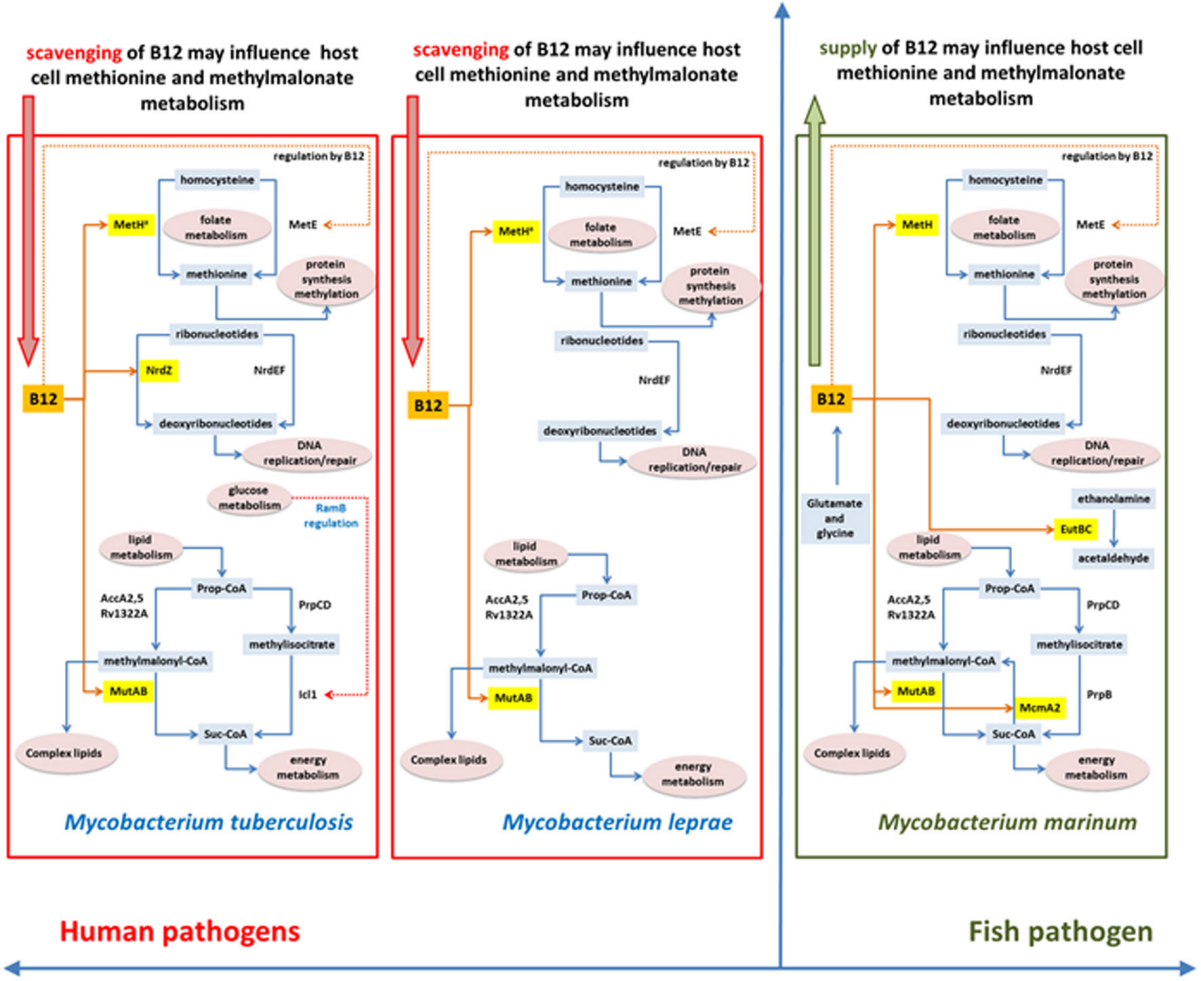
**Differences and similarities between B12-related metabolism between *M. tuberculosis*, *M. leprae* and *M. marinum***.

An important barrier to understanding the biology of infection—and design of effective antibacterial agents—is that the physiology of bacteria growing in culture differs from that of the same bacteria in the host. Direct characterization of the *in vivo* metabolome faces the technical limitation that the vast majority of metabolites are common to both host and pathogen and, at least in the case of tuberculosis, that the heterogeneity of lesions is likely to be mirrored by a corresponding heterogeneity in relevant metabolic phenotypes (Barry et al., 2009). Evolutionary biology offers an alternative approach of using phylogenetic evidence of adaptation to identify areas of metabolism that may play a crucial role in successful pathogenesis. We have used vitamin B12-dependent metabolism in *M. tuberculosis* to explore this strategy, allowing us to sample adaptations to metabolic pathways involved in methionine synthesis, methylmalonate processing, and DNA metabolism. Figure 10 summarizes differences in B12-related metabolism between *M. tuberculosis* and related mycobacterial pathogens *M. marinum* and *M. leprae*.

Phylogenetic analysis highlights a marked difference in the evolution of anaerobic metabolism between closely-related pathogens. While *M. marinum* lacks a class II ribonucleotide reductase and constitutive as well as DosR-regulated enzymes for nitrate respiration, these genes are all present in *M. tuberculosis* together with a fumarate reductase that contributes to reductive operation of the TCA cycle (Rv1552-Rv1555, likely to have been acquired by horizontal transfer) (Watanabe et al., 2011). Further mutations that enhance constitutive expression of the NarG operon and the DosR regulon have been acquired by modern lineages of *M. tuberculosis* (Rose et al., 2013) suggesting an increasing reliance on anaerobic metabolism. This is consistent with inferences from experimental models (Via et al., 2008), but the phylogenetic evidence provides an additional important link to the role of hypoxia in the long-term context of host-pathogen co-evolution in human populations. While *M. marinum* has attractive properties as a surrogate model for tuberculosis drug discovery (Takaki et al., 2012), it would not be an appropriate choice in the case of drugs targeting hypoxic phenotypes. Similar to *M. marinum*, the *M. leprae* genome shows no evidence of a role for anaerobic metabolism.

Phylogenetic analysis highlights a dynamic exchange of genes amongst mycobacteria by horizontal transfer mediated by phages, plasmids, and conjugative elements that is likely to have played a crucial role in pre-adaptation of *M. tuberculosis* to its pathogenic role. While several *M. tuberculosis* virulence determinants can be traced back to horizontal acquisition by the *M. canettii*-like ancestor (Veyrier et al., 2009; Supply et al., 2013; Wang and Behr, 2014), it cannot be assumed that all such acquisitions reflect positive selection—many unique loci may simply be “passing through,” unlinked to functional biology of the host organism. The absence of further gene acquisition during diversification of the *M. tuberculosis* complex is a fundamentally important aspect of the biology of tuberculosis. *M. tuberculosis* is forced to rely on alterations to its existing genetic complement to optimize survival in an evolving human host, and is denied access to mobile drug-resistance elements. It has been proposed that *M. tuberculosis* has adapted to a more virulent phenotype over the course of its co-evolution with human populations (Comas et al., 2013); changes in vitamin B12 availability during the Neolithic transition from predominantly meat-eating hunter gatherers to vegetarian farmers represents one potential stimulus for mycobacterial adaptation.

### Conflict of interest statement

The authors declare that the research was conducted in the absence of any commercial or financial relationships that could be construed as a potential conflict of interest.
